# Physical Activity, Nutritional Habits, and Sleeping Behavior in Students and Employees of a Swiss University During the COVID-19 Lockdown Period: Questionnaire Survey Study

**DOI:** 10.2196/26330

**Published:** 2021-04-13

**Authors:** Jan Taeymans, Eefje Luijckx, Slavko Rogan, Karin Haas, Heiner Baur

**Affiliations:** 1 Department of Health Professions Bern University of Applied Sciences Bern Switzerland; 2 Faculty of Physical Education and Physiotherapy Vrije Universiteit Brussel Brussels Belgium; 3 Akademie für integrative Physiotherapie und Trainingslehre (AfiPT) Grenzach-Whylen Germany

**Keywords:** COVID-19, healthy lifestyle, pandemics, public health, universities

## Abstract

**Background:**

The new coronavirus SARS-CoV-2 led to the COVID-19 pandemic starting in January 2020. The Swiss Federal Council prescribed a lockdown of nonessential businesses. Students and employees of higher education institutions had to install home offices and participate in online lectures.

**Objective:**

The aim of this survey study was to evaluate lifestyle habits, such as physical activity (PA), sitting time, nutritional habits (expressed as median modified Mediterranean Diet Score [mMDS]), alcohol consumption habits, and sleeping behavior during a 2-month period of confinement and social distancing due to the COVID-19 pandemic. Survey participants were students and employees of a Swiss university of applied sciences.

**Methods:**

All students and employees from Bern University of Applied Sciences, Department of Health Professions (ie, nursing, nutrition and dietetics, midwifery, and physiotherapy divisions) were invited to complete an anonymous online survey during the COVID-19 confinement period. Information on the lifestyle dimensions of PA, sitting time, nutritional and alcohol consumption habits, and sleep behavior was gathered using adaptations of validated questionnaires. Frequency analyses and nonparametric statistical methods were used for data analysis. Significance was set at 5% α level of error.

**Results:**

Prevalence of non-health-enhancing PA was 37.1%, with participants of the division of physiotherapy showing the lowest prevalence. Prevalence of long sitting time (>8 hours/day) was 36.1%. The median mMDS was 9, where the maximal score was 15, with participants of the division of nutrition and dietetics being more adherent to a Mediterranean diet as compared to the other groups. Prevalence of nonadherence to the Swiss alcohol consumption recommendations was 8.3%. Prevalence of low sleeping quality was 44.7%, while the median sleeping duration was 8 hours, which is considered healthy for adult populations.

**Conclusions:**

In the group analysis, differences in PA, sitting time, and mMDS were observed between different divisions of health professions as well as between Bachelor of Science students, Master of Science students, and employees. Therefore, public health messages regarding healthy lifestyle habits during home confinement should be more group specific. The results of this study may provide support for the implementation of group-specific health promotion interventions at universities in pandemic conditions.

**Trial Registration:**

ClinicalTrials.gov NCT04502108; https://www.clinicaltrials.gov/ct2/show/NCT04502108

## Introduction

### Background

The World Health Organization declared the new coronavirus SARS-CoV-2 leading to COVID-19 as a pandemic on March 11, 2020 [1]. Then 5 days later, the Swiss Federal Council declared an “extraordinary situation” in terms of the Epidemics Act. Stringent measures were put in place [2]. All nonvital businesses as well as schools of all levels, including universities and universities of applied sciences, had to be closed. To contain the pandemic, the Swiss Federal Council called on members of the public to remain at home in order to keep their distance from others.

This lockdown was immediately instituted by the president of Bern University of Applied Sciences (BFH). Classroom teaching was forbidden. Students and employees had to remain at home, continuing their study and work in home office settings. Lecturers were asked to switch to digitalization to guarantee the continuation of the different educational programs during this second part of the spring 2020 academic semester and upcoming fall 2020-2021 semester [3].

All sports infrastructure in Switzerland was forced to close during this nearly 2-month lockdown period. While regular access to fitness clubs or sports facilities was no longer possible, individual walking, jogging, and cycling, however, was still allowed. Food shops remained open during this period. Citizens were allowed to go outside for food supplies when adhering to the hygiene measures [2].

It can be hypothesized that such severe restrictions may have an influence on healthy lifestyles [4,5]. Some international studies reported on lifestyle changes during home confinement. In Italy, the perception of weight gain was observed in 48.6% of the population and a slight increase in physical activity (PA) has been reported [6]. Another study with data from Western Asia, North Africa, Europe, and other countries revealed that the COVID-19 home confinement has had a negative effect on all levels of PA and an increase in daily sitting time by more than 28% [7].

Hamer et al [8] suggested that an unhealthy lifestyle synonymous with an elevated risk of noncommunicable disease is also a risk factor for COVID-19 hospital admission.

There is limited knowledge on lifestyle habits, such as PA, physical inactivity (ie, sitting time), nutritional and alcohol consumption habits, and sleeping behavior, during an extraordinary period of 2 months’ confinement and social distancing in university students [9-12], and information regarding university employees is even more scarce.

Because of their health profession–specific scholarly knowledge, differences in lifestyle habits between members of the different health profession divisions (eg, between the nutrition and dietetics division and physiotherapy) can be expected. Furthermore, in the Swiss context, Bachelor of Science (BSc) programs of the different health profession divisions are full time while most of the Master of Science (MSc) programs are scheduled as part time, allowing the latter students to combine study and work. Similar to the BSc students, most employees were also confined at home. Thus, it can be hypothesized that differences in lifestyle habits during a lockdown period can be observed between these three groups: BSc students, MSc students, and employees. 

Because this pandemic is caused by a *new* coronavirus lacking vaccination and treatment possibilities, predictions on the development of the pandemic (eg, the rise of a *second wave* during winter) remains difficult. Increased knowledge about lifestyle habits of students and employees of the BFH, Department of Health Professions (BFH-DHP), during such an extreme confinement situation may help heads and deans of academic institutions as well as other decision makers to counsel students and employees during a similar situation or in case of another outbreak in the future. However, due to the uniqueness of this COVID-19 crisis and its societal impact, such knowledge is currently lacking.

### Objective

This study evaluated differences in lifestyle habits, such as PA, sitting time, nutritional and alcohol consumption habits, and sleeping behavior, during COVID-19 home confinement (spring 2020) with social distancing between BSc students, MSc students, and employees as well as between the four health profession divisions (ie, nutrition and dietetics, midwifery, nursing, and physiotherapy) of BFH-DHP, Switzerland.

Delineated research questions were as follows: (1) Are there lifestyle differences between the four groups of health profession divisions (ie, nutrition and dietetics, midwifery, nursing, and physiotherapy) during lockdown home confinement? and (2) Are there lifestyle differences between BSc students, MSc students, and employees during lockdown home confinement?

## Methods

This survey was conducted as an interdisciplinary collaboration between faculty members of the division of Physiotherapy and the division of Nutrition and Dietetics. A protocol of this observational study has been published elsewhere [13]. Here, a brief summary of the methods is presented.

### Research Design

For this study, a self-reported electronic survey was conducted within the 2020 COVID-19 strict lockdown period assessing PA, sitting time, nutritional and alcohol consumption habits, and sleeping habits in students and employees of BFH-DHP, Switzerland.

Ethical issues were considered. Prior to the start of this survey, the dean of BFH-DHP was informed and approved this study. In the introductory section of the survey, eligible staff and students were informed that the survey was voluntary and anonymous, that no medical data will be asked for, and that they could contact the researchers for any information or further questions. Finally, it was explained that by filling out the survey and resubmitting it to the system, they explicitly gave their informed consent. The EvaSys software (Electric Paper Evaluationssysteme GmbH) does not allow for any personal tracing of the respondents. The study was submitted to the Ethical Committee of Canton Bern. The Ethical Committee declared that this anonymous survey without medical data did not need to undergo a full approval procedure (KEK Bern, Req-2020-00909) because it does not fall under the regulations of the *Federal Act on Research involving Human Beings* in Switzerland. The survey has been registered at ClinicalTrials.gov (NCT04502108).

### Study Participants

All students (n=1300; 88.0% [1144/1300] females and 12.0% [156/1300] males) enrolled in BSc or MSc study programs in the field of nursing, nutrition and dietetics, midwifery, and physiotherapy, as well as all academic and nonacademic employees (n=268) from BFH-DPH, were eligible and were invited to volunteer in this electronic survey.

Independent measures were BSc students, MSc students, and employees as well as the four health profession divisions.

### Data Collection, Data Management, and Data Analysis

The survey was sent via the institute’s email system to all staff and students on May 5, 2020, and remained open until May 15, 2020, to ensure a full COVID-19 confinement snapshot. A brief introduction section prior to the different questionnaires explained the objective of the survey. Automated reminders were sent two times during this time slot.

Data collection was performed anonymously using the *evaluation system software* of BFH-DPH, EvaSys. A standardized questionnaire was developed within the EvaSys framework, including validated tools to assess PA and sitting time (ie, the International Physical Activity Questionnaire–Short Form [IPAQ-SF]) [14] and to evaluate nutritional habits (ie, a Swiss adaptation of the brief Mediterranean Diet Screener [bMDSC]) [15]. Questions on alcohol consumption and sleeping behavior were added to the survey, while, for reasons of anonymity, questions on socioeconomic status were omitted. Lifestyle habits under evaluation were assessed during the 7 days prior to filling out the survey. A complete description of the data management, cleansing, and analysis can be consulted in Multimedia Appendix 1. A brief description follows.

The IPAQ-SF assesses PA undertaken across four domains, including leisure time PA, domestic and gardening activities, work-related PA, as well as transport-related PA. The IPAQ-SF evaluates three specific types of PA (ie, walking, moderate-intensity PA, and vigorous-intensity PA) undertaken in these four domains during the previous 7 days. Time spent in the three types of PA was calculated and expressed in minutes. The IPAQ-SF algorithm was used to transform the continuous data into categorical data (ie, *low*, *moderate*, and *high, health-enhancing* PA) [14]. A reliability study of the IPAQ-SF including 178 Swiss volunteers found fair to good reliability with Spearman correlation coefficients of 0.54 for total PA (MET [metabolic equivalent] min/week) and 0.60 for sitting [16].

The analysis of sitting time during the previous 7 days was also conducted following the IPAQ-SF guidelines.

A Swiss adaptation of the bMDSC was used to assess nutritional habits and adherence to the Mediterranean diet, which has been proposed as a healthy eating pattern because of its high content of antioxidant food items. Volunteers were asked to report their adherence to a recommended consumption frequency of 15 selected food items during the preceding 7 days. Answer categories were *yes* or *no*. Healthy items scored 1 if answered with *yes* and 0 otherwise, while unhealthy items were reverse coded. Scores were summed to calculate a modified Mediterranean Diet Score (mMDS). The maximal score is 15, with higher scores indicating better adherence to the Mediterranean diet. A validation study including 102 participants reported an intraclass correlation coefficient of 0.4 (*P*<.001) between the mMDS derived from the bMDSC and a 24-hour recall index. Reported limits of agreements were 59% and 144%. The authors concluded that the bMDSC is a valid tool for rapid assessment of dietary quality [15].

Daily wine, beer, and spirits (ie, liquor) consumption during the preceding 7 days was given in units (ie, glasses). While there is evidence that drinking patterns may matter more than the type of alcohol [17,18] consumption itself, it has also been proposed that adherence to a Mediterranean diet with a moderate wine intake during meals could explain the observed lower prevalence of cardiovascular disorders in Southern Europe as compared to Northern Europe. Furthermore, wine and spirits are more expensive than beer [19]. Therefore, in addition to alcohol intake frequency data, this study wanted to differentiate between the types of alcohol consumed.

Sleeping behavior during the preceding 7 days was given as time to go to bed and wake-up time. Sleeping duration was calculated as the difference between these two times. Quality of sleep was asked to be rated as *no sleeping problems*, *sleeping quality could be improved*, or *important sleeping problems*.

Data management was conducted on the institutional server while data cleansing was performed by one researcher (JT) to check for incompatibilities and to control plausibility of the data (eg, range checks). The IPAQ-SF data cleansing rules were followed: participants with incomplete data or who mentioned “don’t know” were removed from the analysis [14].

### Statistical Analyses

Frequency analyses and nonparametric statistics were used to report the results of this survey. For the descriptive analyses, central tendencies were expressed as medians, while variation was reported using the 25^th^ and 75^th^ percentiles and IQRs. Kruskal-Wallis tests and Whitney *U* tests with post hoc Bonferroni corrections were used to assess differences between independent groups (ie, between BSc and MSc students and employees as well as between members of the four divisions). Results are presented as frequency tables or as figures with box plots.

Data were prepared in Excel 2018 (Microsoft Corporation) but imported into SPSS Statistics for Windows, version 26.0 (IBM Corp), for statistical analyses. Statistical significance was set at the 5% level of error.

## Results

### Overview

A total of 821 participants (BSc students: 616/821, 75.0%; MSc students:100/821, 12.2%; employees: 105/821, 12.8%) volunteered for this online survey. Students’ and employees’ response rates were 55.1% and 39.2%, respectively. Respondents were affiliated with the divisions of nutrition and dietetics (119/821, 14.5%), midwifery (109/821, 13.3%), nursing (309/821, 37.6%), and physiotherapy (284/821, 34.6%).

Because incomplete files were excluded from the different analyses, the sample sizes were reduced to 650 out of 821 (79.2%) respondents for the PA analysis, 761 out of 821 (92.7%) for the sitting time analysis, 771 out of 821 (93.9%) for the nutritional habits analysis, 815 out of 821 (99.3%) for the alcohol consumption analysis, and 796 out of 821 (97.0%) for the sleeping time and quality analysis.

### Physical Activity

In this sample of 650 respondents, never engaging in vigorous PA, moderate PA, or walking during the preceding 7 days was reported by 67 (10.3%), 44 (6.8%), and 18 (2.8%) participants, respectively. A total of 4 volunteers did not participate in any of the three PA types. The median MET minutes per week score was 3447 (IQR 2117-5396). Of the 650 volunteers, 30 (4.6%) were classified as *low*, 211 (32.5%) as *moderate*, and 409 (62.9%) as *high* for PA.

**Figure 1:**
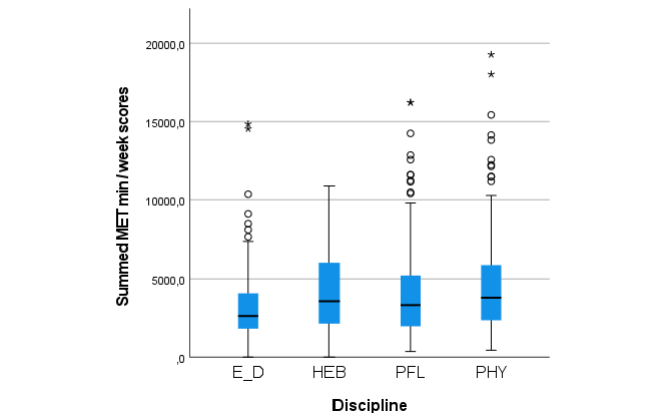
Box plots of the physical activity scores (summed metabolic equivalent [MET] min/week scores) per health profession division of a Swiss university of applied sciences during the spring 2020 COVID-19 lockdown. Whiskers indicate 1.5 × IQR unless truncated at the lowest score. Asterisks and circles represent values outside this range. E_D: nutrition and dietetics; HEB: midwifery; PFL: nursing; PHY: physiotherapy.

Figure 1 presents the box plot of the summed MET minutes per week scores for the four different health profession divisions (ie, nutrition and dietetics, midwifery, nursing, and physiotherapy). Participants of the division of nutrition and dietetics showed a lower median summed MET minutes per week score compared to that of the division of physiotherapy (*P*=.001). The other observed differences between divisions were not statistically significant (*P*>.05). Calculated values of the box and whisker plots are presented in Multimedia Appendix 2.

Table 1 shows the absolute and relative frequencies of the categories *low*, *moderate*, and *high, health-enhancing* PA in the groups of the four health profession divisions. The highest relative frequency of *high* PA was found in the participants from the division of physiotherapy (175/236, 74.2%), while the highest relative frequency of *low* PA was observed in the volunteers from the division of midwifery (8/79, 10.1%). Table 1 depicts the absolute and relative frequencies of the classifications in *low*, *moderate*, and *high* PA for the 650 participants of the two student groups (ie, BSc and MSc) and the employee group. The highest relative frequency of *high* PA was found in the group of MSc students (54/79, 68.4%), while the highest relative frequency of *low* PA was observed in the employee group (9/91, 9.9%)

**Table 1:** Categorized physical activity data of the preceding 7 days of 650 students and employees in four divisions of a Swiss university of applied sciences during the spring 2020 COVID-19 lockdown.

Participant group			Physical activity level per group, n (%)						
			High		Low		Moderate		Total
**Division**									
	Nutrition and dietetics	49 (49)		3 (3)		47 (48)		99 (100)	
	Midwifery	47 (60)		8 (10)		24 (30)		79 (100)	
	Nursing	138 (58.5)		12 (5.1)		86 (36.4)		236 (100)	
	Physiotherapy	175 (74.2)		7 (3.0)		54 (22.9)		236 (100)	
**Students or employees**									
	Bachelor of Science students	307 (64.0)		20 (4.2)		143 (29.8)		480 (100)	
	Master of Science students	54 (68)		1 (1)		24 (30)		79 (100)	
	Employees	48 (53)		9 (10)		34 (37)		91 (100)	

**Figure 2:**
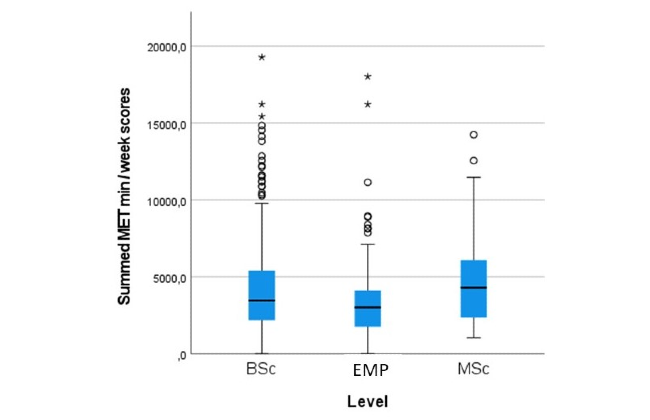
Box plots of the physical activity scores (summed metabolic equivalent [MET] min/week scores) for students and employees (n=650) of a Swiss university of applied sciences (health professions) during the spring 2020 COVID-19 lockdown. Whiskers indicate 1.5 × IQR unless truncated at the lowest score. Asterisks and circles represent values outside this range. BSc: Bachelor of Science students; EMP: employees; MSc: Master of Science students.

Figure 2 shows the box plot of the summed MET minutes per week scores for the BSc student, MSc student, and employee groups. The employee group showed a lower median summed MET minutes per week score compared to the group of MSc students (*P*=.002) and the group of BSc students (*P*=.04). There was no difference in the median MET minutes per week scores between the BSc and MSc student groups (*P*=.12). Calculated values of box and whisker plots are presented in Multimedia Appendix 2.

### Sitting Time

**Figure 3:**
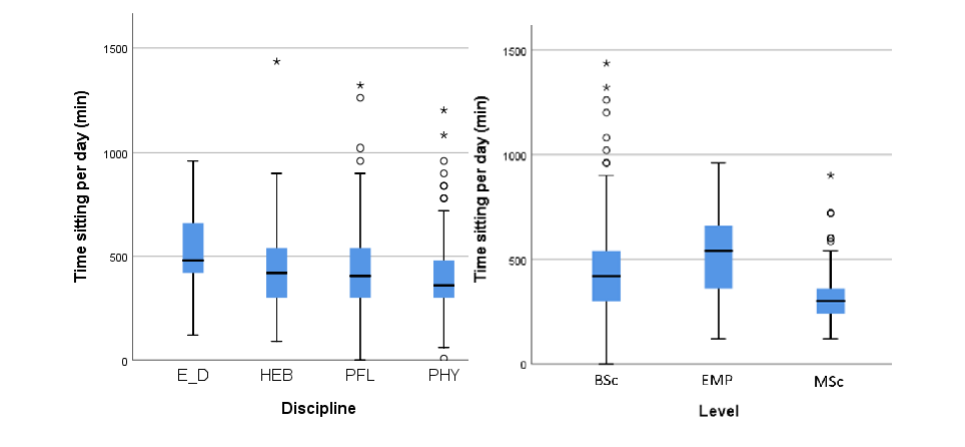
Box plots showing the daily sitting times (in minutes) among students and employees (n=761) from four divisions of a Swiss university of applied sciences during the spring 2020 COVID-19 lockdown. Left: daily sitting times per health profession division. Right: daily sitting times per group of students and employees. Whiskers indicate 1.5 × IQR unless truncated at the lowest score. Asterisks and circles represent values outside this range. BSc: Bachelor of Science students; E_D: nutrition and dietetics; EMP: employees; HEB: midwifery; MSc: Master of Science students; PFL: nursing; PHY: physiotherapy.

A total of 761 out of the 821 respondents (92.7%) were included in this analysis. Median sitting time was 420 minutes per day (IQR 300-540). Figure 3 (left) depicts the box plot of the daily sitting time for members of the four different health profession divisions. Participants from the nutrition and dietetics division had higher median daily sitting time values compared to those from the other health profession divisions (all comparisons *P*<.001). Figure 3 (right) presents the box plot of daily sitting time values for the BSc student, MSc student, and employee groups. Employees had a higher median daily sitting time value compared to values of the BSc and MSc students (all comparisons *P*<.001). BSc students showed a higher median daily sitting time value compared to that of the MSc students (*P*<.001). Calculated values of the box and whisker plots are presented in Multimedia Appendix 2.

### Nutritional Habits

A total of 771 out of the 821 respondents (93.9%) could be included in this analysis. The median mMDS in this sample was 11 (IQR 9-12). The lowest mMDS observed in this sample was 2 (n=1), while 8 persons were fully adherent to the Mediterranean diet (mMDS=15).

**Figure 4:**
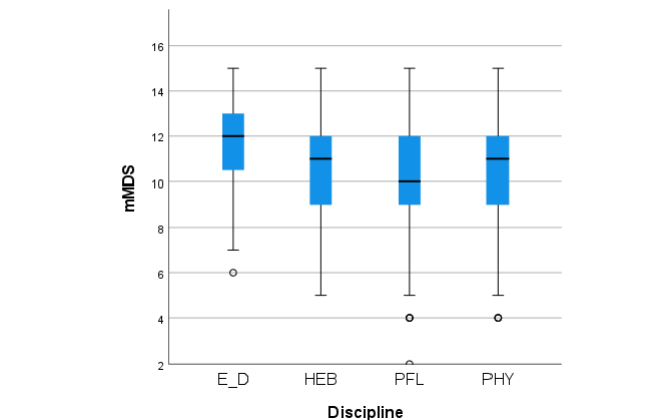
Box plots of the eating habits, measured using the modified Mediterranean Diet Score (mMDS), of students and employees (n=771) in each health profession division of a Swiss university of applied sciences during the spring 2020 COVID-19 lockdown. Whiskers indicate 1.5 × IQR unless truncated at the lowest score. Circles represent values outside this range. E_D: nutrition and dietetics; HEB: midwifery; PFL: nursing; PHY: physiotherapy.

Figure 4 depicts the box plot of the mMDS values for members of the four different health profession divisions. Participants from the division of nutrition and dietetics had a higher median mMDS compared to those from the divisions of nursing and physiotherapy (both comparisons *P*<.001) or the division of midwifery (*P*=.03). The median mMDS of the participants from the division of midwifery was higher compared to that of the participants from the division of nursing (*P*=.047). Calculated values of the box and whisker plots are presented in Multimedia Appendix 2.

No differences were found between BSc students, MSc students, and employees (*P*=.17).

### Alcohol Consumption

A total of 815 out of the 821 respondents (99.3%) were included in this analysis. Table 2 shows the absolute and relative frequencies of the different types of alcohol consumption among these 815 participants. Over 80% of the volunteers reported no wine or beer consumption over the preceding 7 days, while nearly 97% reported no liquor or spirits consumption over the same period. Around 18% of the respondents adhered to the Mediterranean diet guideline of 2 units of wine per day. Out of 815 participants, 23 (2.8%) reported daily combinations of the different types of alcohol consumption above 3 units per day. Out of 815 participants, 2 (0.2%) reported an excessive alcohol consumption of more than 7 units of all types of drinks per day.

**Table 2:** Different types of alcohol consumption over the preceding 7 days by 815 students and employees in four divisions of a Swiss university of applied sciences during the spring 2020 COVID-19 lockdown.

Type of alcohol	Units of drinks consumed per day by participants (N=815), n (%)					
	0	1	2	3	4-7	>7
Wine	651 (79.9)	134 (16.4)	12 (1.5)	0 (0)	11 (1.3)	7 (0.9)
Beer	685 (84.0)	109 (13.4)	14 (1.7)	4 (0.5)	1 (0.1)	2 (0.2)
Liquor or spirits	788 (96.7)	20 (2.5)	3 (0.4)	2 (0.2)	0 (0)	2 (0.2)

No differences in alcohol consumption were observed between the four health profession division groups (*P*>.05). Similarly, no group differences between the BSc student, MSc student, and employee groups were found (*P*>.05).

### Sleeping Behavior

A total of 796 out of the 821 respondents (97.0%) were included in this analysis. Of those, 44 (5.5%) reported poor sleeping quality, 312 (39.2%) found that sleeping quality could be improved, while 440 (55.3%) reported good sleeping quality.

No differences in sleeping quality were observed between the four health profession division groups (*P*>.05). Similarly, no group differences between the BSc student, MSc student, and employee groups were found (*P*>.05).

In this sample, 253 out of 796 respondents (31.8%) reported going to bed at 11 PM. Only 1 volunteer (0.1%) mentioned that their bedtime was at 7:30 PM, while another respondent (0.1%) went to bed at 3:30 AM. A total of 176 persons (22.1%) reported waking up at 7 AM. Only 1 participant (0.1%) mentioned a wake-up time of 4 AM, while another (0.1%) mentioned not getting out of bed before noon. Median sleep duration in this sample was 8 hours (IQR 7.8-9.0).

No group differences for bedtime, wake-up time, and sleep duration between the four different health profession division groups were found (all comparisons *P*>.05).

**Figure 5:**
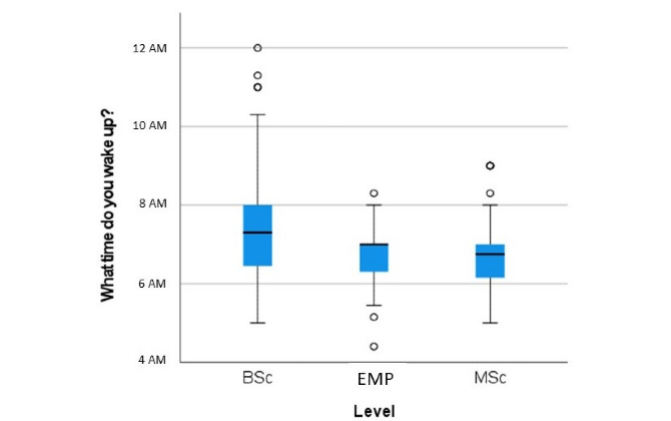
Box plots of wake-up time of students and employees (n=796) from the Bern University of Applied Sciences, Department of Health Professions during the spring 2020 COVID-19 lockdown. Whiskers indicate 1.5 × IQR unless truncated at the lowest score. Circles represent values outside this range. BSc: Bachelor of Science students; EMP: employees; MSc: Master of Science students.

No group differences between BSc students, MSc students, and employees were found for bedtime (*P*=.15). Figure 5 presents the box plot of the wake-up times for those three groups. Median wake-up time was later in the BSc student group compared to the MSc student and employee groups (both *P*<.001). Calculated values of the box and whisker plots are presented in Multimedia Appendix 2.

### Availability of Data and Material

The data set used during this study is available from the corresponding author on reasonable request.

## Discussion

### Principal Findings

This study found differences in PA, sitting time, nutritional and alcohol consumption habits, and sleeping behavior between BSc students, MSc students, and employees as well as between the four health profession divisions (ie, nutrition and dietetics, midwifery, nursing, and physiotherapy) of a university of applied sciences in Switzerland during COVID-19 home confinement (spring 2020) with social distancing.

In the same period, similar initiatives in the general population were launched by other institutions. For example, an Italian survey including 398 university students used the International Physical Activity Questionnaire to assess PA and sedentary behavior during COVID-19 lockdown in spring 2020. Lockdown sedentary behavior was greater than before lockdown (*P*=.003). While closure of the university increased sedentary behavior across the sample, it only decreased PA in participants who were the most active before lockdown [20].

In this study, response rates of students (55.1%) and employees (39.2%) were higher than expected in the study protocol (30%) [13]. Participants from the nursing and physiotherapy divisions together represented 72.2% of the total respondents (593/821). Both divisions also contribute the largest numbers of students and employees in the total BFH-DHP population. BSc students were 6 times more represented in this study sample than MSc students or employees. Undergraduate students also represent the highest number in the total BFH-DHP population.

During the 2020 COVID-19 confinement period, about 90% of the 650 respondents that could be included in the PA analysis were engaging in one of the three types of PA (ie, vigorous PA, moderate PA, or walking) during the preceding 7 days before filling out the survey questionnaires. On an individual level, 4 participants reported never having participated in such activities over the previous 7 days. The median summed MET minutes per week score was 3447 but with large variation (IQR 2117-5396). The IPAQ-SF interpreted the results from the perspective of health-enhancing effects: 4.6% of the respondents were classified as *inactive*, 32.5% as *minimally active*, and 62.9% as *health-enhancing physically active*. Persons classified as belonging to the latter group participated in PA bouts with evidence of health-enhancing effects [14]. These results are lower than those from the data from the Swiss Health Survey 2017, where the proportion of trained and sufficiently active people who met the PA recommendations was 76% [21]. Only participants from the division of physiotherapy (74.2%) met these criteria of health-enhancing PA during confinement. Participants from the division of nutrition and dietetics showed the lowest median PA level. The proportion of inactive or insufficiently active persons in this study is comparable with the Swiss Health Survey. The observed difference between these two groups was 85 MET minutes representing a short walking tour of 15 minutes for 5 days a week. When summed up over time, even small differences may become clinically relevant. The importance of PA is known. Active muscles produce chemicals that improve immune functioning, which in turn reduces the extent of infections and decreases inflammation, and these are the main causes of the lung damage from SARS-CoV-2 infection [22].

Students had a higher median PA level compared to employees. It is interesting to note that in this specific sample of health profession students and employees, 37.1% were not participating at a health-enhancing PA level. Being a health profession student or health professional may not always have a protective effect against unhealthy lifestyle habits [23-25]. It is encouraging to observe that participants from the division of physiotherapy—those who are, or have been, trained to become movement science experts—showed the highest median PA levels. It can be argued that participants in this specific sample have, on average, a higher health literacy than their peers from other faculties and, thus, an underestimation of the number of participants not adhering to a health-enhancing PA level cannot be excluded at the total population level of the university of applied sciences.

Results of this survey suggest that incentives organized by universities may be needed to empower students and employees more specifically. For example, action plans for workplace health promotions may be developed with a special focus on digital dissemination paths to reach students and employees in their home office settings.

Median sitting time in this sample during the preceding 7 days was 7 hours per day, with a prevalence of long sitting time (>8 hours/day) of 36.1%, which is higher compared to the Swiss data, where 25% of the employed persons sat for more than 8 hours a day [21]. The prevalence of long sitting time in university students under normal, nonconfinement conditions is high and its effect on cognition and academic performance is not well studied yet. A Spanish study including 372 undergraduate university students concluded that introducing health promotion programs into university settings to replace leisure sitting time with moderate PA may contribute to enhanced student performance [26]. Participants from the nutrition and dietetics division showed the highest median sitting time (8 hours/day) compared to their peers from the other health disciplines. The median sitting time for employees during the COVID-19 confinement period was more than 8 hours. The observed higher sitting time for BSc students as compared to their peers at the MSc level can be, at least partially, explained by the type of study program. While most BSc programs are full time, most MSc programs at BFH-DHP are part time. Even under strict confinement conditions, most graduate students were still working in the health care sector and, hence, this might have resulted in less sitting time as compared to their undergraduate peers.

The findings for PA and sitting time may have important public health implications. For example, health promotion campaigns to increase PA and reduce physical inactivity should focus on employees and students, especially those in the nutrition and dietetics division. They should be empowered to participate in health-enhancing PAs and to reduce daily sitting time in periods with strict confinement conditions when fitness centers and other sports facilities are closed. This study gives an opportunity to implement a module on healthy PA in the curriculum of the nutrition and dietetics division, and possibly in those for nursing and midwifery, of BFH-DHP. Furthermore, the BFH-DHP team for workplace health promotion might plan a similar module specifically for employees. Measures should be instigated to detect the very small group of physically inactive students and employees and to increase their health literacy on the negative effects of a totally sedentary lifestyle. Notwithstanding regular modifications of the curriculum, ad hoc action plans for an acute pandemic situation consisting of mainly digital distribution pathways may help and empower students and employees during similar lockdown situations.

Adherence to the Mediterranean diet during the preceding 7 days, as a reference for a healthy eating pattern [15] in this study, yielded a median mMDS of 11 (IQR 9-12) where the maximal score was 15. A total of 142 out of the 771 respondents (18.4%) included in the nutritional analyses showed low adherence (25^th^ percentile <9) to a healthy eating pattern during the confinement period. Undesirable changes to diet patterns have the potential to persist for some time, even after isolation measures are eased [27]. Participants from the nutrition and dietetics division adhered best to the healthy eating pattern, while participants from the nursing division adhered least well to the Mediterranean diet. It is encouraging to observe that participants from the nutrition and dietetics division—those who are, or have been, trained to become expert dietitians—showed the highest median mMDS. This indicates that they are adhering to a healthy eating pattern even under strict confinement conditions.

In this sample 96.3%, 97.4%, and 99.1% of the volunteers reported being abstinent or not drinking more than 1 unit of wine, beer, or liquor and spirits daily during the preceding 7 days. They adhered to the guidelines of the Swiss Federal Commission on Alcohol Issues for healthy adult females [28]. Following those Swiss guidelines, healthy adult males would be allowed to drink 2 units of alcoholic beverages daily. Alcohol consumption was evenly distributed across the participants of the different health profession divisions and between student study level and employee groups. The prevalence of moderate and high-risk alcohol drinkers in this study was much lower than in the German study of Keller et al [24], who reported 65% binge drinking in first-year university students. It is encouraging to observe the high prevalence of alcohol abstinence and healthy alcohol consumption habits in participants of the different health profession divisions of BFH-DHP, even under strict confinement conditions. However, on the individual level, 68 out of the 815 volunteers (8.3%) included in the alcohol consumption analysis reported daily alcohol intake of more than 1 unit, while 0.9% reported drinking more than 3 units per day of combinations of the different types of alcohol. These participants did not adhere to the Swiss healthy alcohol drinking guidelines for adult females [28]. It is impossible to check if the same 2 persons who reported a daily alcohol intake of more than 7 units of three types of alcohol are binge drinkers or simply reported their true consumption inaccurately.

Results of this study on nutritional habits and alcohol consumption may have important public health implications. When preparing health promotion campaigns to improve adherence to a more healthy eating pattern, the leaders of universities with different health profession divisions should primarily focus on those students and employees who are not enrolled at the division of nutrition and dietetics to empower them to practice healthier eating patterns, especially during a confinement period. This could be achieved by implementing a module on healthy eating habits in the curriculum of the divisions of midwifery, nursing, and physiotherapy. Furthermore, the team for workplace health promotion of such universities might plan such a module specifically for employees. Measures should be installed to detect the small group of students and employees with low to very low adherence to the Mediterranean diet and to increase their health literacy on the negative effects of an unhealthy eating pattern. Health promotion campaigns to strengthen the observed healthy attitude toward alcohol consumption in the majority of participants should focus on all students and employees; in addition, measures should be put in place to detect the small group of students and employees with unhealthy drinking behavior to increase their health literacy on the negative health and social effects of alcohol abuse, especially during such a confinement period.

During the confinement period and during the 7 days that preceded participation in this survey, the prevalence of poor sleeping quality or sleeping quality that could be improved was 44.7%. This is consistent with a study by Salehinejad et al [29] that showed that participants reported significantly poorer sleep quality in home quarantine during the COVID-19 crisis compared to the prequarantine time. For sleeping quality, no group differences between the participants of the different health profession divisions or between the students at different study levels and employees were found. The median sleeping duration in this sample was 8 hours, which represents adherence to healthy sleep guidelines [30]. Again, no group differences were observed. Prevalence of short sleep duration, defined as less than 7 hours of sleep in a 24-hour period [31], was 5.5%. This number is low compared to the prevalence of short sleep duration of 57.8% observed in 52,256 middle and high school students in the United States [32]. There is evidence that short sleep duration is associated with risk factors such as obesity, diabetes, mental health, and poor academic performance [32].

The observed later median wake-up time in the BSc students as compared to the MSc student group and the employee group could, at least partially, be explained by the fact that most BSc programs are full time while most MSc programs at BFH-DHP are part time, allowing MSc students to go to work.

### Limitations

This study was conducted in a highly specific sample of members of the four divisions of different health professions at BFH-DHP in Switzerland. Generalizability to other universities or faculties may be hampered. Furthermore, this study evaluated the prevalence of risk factors in this population of university students and employees during the confinement period only. Therefore, a comparison with the preconfinement period cannot be made. To keep the questionnaire short and to guarantee anonymity, other important risk factors (eg, smoking status and stress status), demographic data (eg, age, sex, and living situation), and socioeconomic status data were omitted, making it impossible to correct for potential confounding factors. Socioeconomic status is indeed an important risk factor. Gallo et al [27] found that university students who had at least one graduate parent were more likely to achieve recommended levels of PA even during the lockdown as compared to their peers who had no graduate parent [10]. In self-reported surveys, a social desirability bias cannot be excluded.  This is a special type of response bias describing a tendency of survey respondents to answer questions in a manner that will be viewed favorably by others and may lead to underreporting of unhealthy lifestyle habits and overreporting of healthy lifestyle habits [33]. This bias makes comparisons and interpretation of average tendencies difficult and has been well described in students of nutrition and dietetics [34]. It can be assumed that the issue of social desirability bias applies also for students and employees of other health professions. Another limitation of this study may be the lack of information about the current health status of the participants. Acute illness around the time of the survey may have interfered with usual PA levels or with nutritional and sleeping habits. Finally, a recently published meta-analysis on the validity of the IPAQ-SF concluded that there is but weak evidence to support the IPAQ-SF for the measurement of absolute or relative PA, yet only one of the 23 included studies compared the IPAQ-SF with the doubly labeled water technique as “criterion gold-standard” [35].

### Conclusions

This survey described PA, sitting time, nutritional and alcohol consumption habits, and sleeping behavior of students and employees of a university of applied sciences during the 2020 COVID-19 confinement in Switzerland.

Results of this survey may help to make leaders of universities aware of the burden and the clustering of unhealthy lifestyle habits in students and employees during such a confinement period. Action plans are needed for health promotion campaigns for students and employees to be better prepared if a similar confinement period is imposed in the future. The findings of this study allow group-specific recommendations to be made: health promotion campaigns to increase PA and reduce physical inactivity should focus on students and employees, especially those in the nutrition and dietetics division, while healthy eating campaigns should primarily focus on those students and employees who are not enrolled in the division of nutrition and dietetics to empower them to practice healthier eating patterns, especially during a confinement period.

## Appendix

Multimedia Appendix 1 Data collection, data management, and data analysis.

Multimedia Appendix 2 Calculated values for box and whisker plots for Figures 1 to 5 of the manuscript.

## Abbreviations

BFH: Bern University of Applied Sciences
BFH-DHP: Bern University of Applied Sciences, Department of Health Professions
bMDSC: brief Mediterranean Diet Screener
BSc: Bachelor of Science
IPAQ-SF: International Physical Activity Questionnaire–Short Form
MET: metabolic equivalent
mMDS: modified Mediterranean Diet Score
MSc: Master of Science
PA: physical activity
