# Supplementary material for: Physical Activity, Nutritional Habits, and Sleeping Behavior in Students and Employees of a Swiss University During the COVID-19 Lockdown Period: Questionnaire Survey Study
Source: JMIR Public Health Surveill. 2021 Apr 13;7(4):e26330. doi: 10.2196/26330 (PMC8045773; doi:10.2196/26330)
Supplement: Multimedia Appendix 1 [file publichealth_v7i4e26330_app1.docx]

Multimedia Appendix 1

Data collection, data management and data analysis

The survey was sent via the Institute’s e-mail system to all staff and students on Mai 5^th^ and remained open until Mai 15^th^ 2020, to ensure a full COVID-19 confinement snapshot. A brief introduction section prior to the different questionnaires explained the objective of the survey. Automatized reminders were sent two times during this timeslot.

Data collection was performed anonymously using the “evaluation system software” of BFH-DPH (EvaSys, Electric Paper, Evaluationssysteme GmbH, Lüneburg, Germany). A standardized questionnaire was developed within the EvaSys-framework including validated tools to assess PA and sitting time (i.e. the International Physical Activity Questionnaire Short Form (iPAQ-SF) [1] and to evaluate nutritional habits (i.e. A Swiss adaptation of the brief Mediterranean diet screener (bMDSC)) [2]. Questions on alcohol consumption and sleeping behavior were added to the survey while for reasons of anonymity questions on socio-economic status were omitted.

iPAQ-SF assesses PA undertaken across four domains including leisure time PA, domestic and gardening activities, work-related PA as well as transport-related PA. The iPAQ-SF evaluates three specific types of PA (i.e. walking, moderate-intensity and vigorous-intensity PA) undertaken in these four domains. Time spent in the three types of PA was calculated and expressed in minutes. Times lower than 10 minutes spent on the different types of PA were recoded to zero. Walking, moderate and vigorous PA time values exceeding 180 minutes were truncated to be equal to 180 minutes as suggested by the iPAQ-SF analysis guidelines while iPAQ-SF algorithm was used to transform the continuous data into categorical data (i.e. “low”, “moderate” and “high, health-enhancing” PA) [1]. A reliability study of iPAQ-SF including 178 Swiss volunteers found fair to good reliability with Spearman correlation coefficients of 0.54 for total PA (MET-min.week^-1^) and 0.60 for sitting [3].

The analysis of sitting time was also conducted following the iPAQ guidelines.

A Swiss adaptation of the brief Mediterranean diet screener (bMDSC) was used to assess nutritional habits and adherence to the Mediterranean diet, which has been proposed as a healthy eating pattern because of its high content of anti-oxidant food items. Volunteers were asked to report their adherence to a recommended consumption frequency of fifteen selected food items during the preceding year: (i) use of olive oil for cooking; (ii) use of olive oil as salad dressing; (iii) daily intake of cooked vegetables; (iv) daily intake of raw vegetables; (v) daily intake of fruits; (vi) daily intake of red meat or sausages; (vii) intake of butter and bread for breakfast or diner; (viii) intake of soft-drink or sweetened drinks yesterday; (ix) paying attention to consume nutritional fibers; (x) weekly intake of legumes, chickpeas and beans (xi) intake of fish one to two times per week; (xii) nuts consumption at least at three days per week; (xiii) prefer white meat over read meat; (xiv) intake of rice or pasta with vegetable sauce is part of the diet; (xv) consumed French fries yesterday. Answering categories were “Yes” or “No”. All items, except items (vi), (vii), (viii) and (xv) scored “1” if answered with “Yes” and “0” otherwise, while questions (vi), (vii), (viii) and (xv) were reversed coded (i.e. score “1” and “0” if answered with “No” and “Yes” respectively). Scores were summed to calculate a modified Mediterranean diet score (mMDS). Maximal score is 15 with higher scoring indicating a better adherence to the Mediterranean diet. A validation study including 102 participants reported an ICC of 0.4 (*p* < .001) between mMDS derived from the bMDSC and a 24-hours recall index. Reported limits of agreements were 59% and 144%. Authors concluded that the bMDSC is a valid tool for rapid assessment of dietary quality [2].

Daily wine, beer and spirits (liquor) consumption was asked in units (glasses).

Sleeping behavior was asked as time to go to bed and wake-up time. Sleeping duration was calculated as the difference between these two times. Quality of sleep was asked to be rated as “No sleeping problems”, “Sleeping quality could be improved” or “Important sleeping problems”.

Data management was conducted on the institutional server. Once participants submitted their survey, the EvaSys system automatically stored data as comma separated variables (.cvs) files for students and employees of the four different divisions (i.e. nutrition and dietetics, midwifery, nursing, physiotherapy) at BFH-DHP. Data files were imported and merged to one single data file in Excel (Microsoft Corporation, 2018).

Data cleansing was performed by one researcher (JT) to check and solve for example dot-comma decimal signs incompatibilities and to control plausibility of the data (e.g. range checks). The International Physical Activity Questionnaire Short Form (iPAQ-SF) date cleansing rules were followed strictly: participants with incomplete (missing) data or who mentioned “don’t know” where removed from the analysis [1].

Data analyses were performed in two steps. First, PA, sitting time, nutritional habits, alcohol consumption and sleeping habits data were analyzed separately. Second, an explorative correlational analysis was conducted including only volunteers with a complete set of data for all lifestyle habits under evaluation. Subgroup analyses were conducted to compare differences in the outcomes between health profession divisions or between study levels (i.e. BSc, MSc) and employees.

Reference

1. IPAQ-group. Guidelines for Data Processing and Analysis of the International Physical Activity Questionnaire (IPAQ). 2005; Available from: <https://sites.google.com/site/theipaq/>.

2. Schroder H, Benitez-Arciniega A, Soler C, Covas M-I, Baena-Díez J, Marrugat J. Validity of two short screeners for diet quality in time-limited Settings. Public health nutrition. 2011 08/23;15:618-26. doi: 10.1017/S1368980011001923.

3. Mader U, Martin BW, Schutz Y, Marti B. Validity of four short physical activity questionnaires in middle-aged persons. Med Sci Sports Exerc. 2006 Jul;38(7):1255-66. PMID: 16826022. doi: 10.1249/01.mss.0000227310.18902.28.
