# Supplementary material for: Physical Activity, Nutritional Habits, and Sleeping Behavior in Students and Employees of a Swiss University During the COVID-19 Lockdown Period: Questionnaire Survey Study
Source: JMIR Public Health Surveill. 2021 Apr 13;7(4):e26330. doi: 10.2196/26330 (PMC8045773; doi:10.2196/26330)

| Sum_MET_min_(Figures 1 and 2 in full text) | | |  | | |  | | |
| --- | --- | --- | --- | --- | --- | --- | --- | --- |
|  | E_D | HEB | PFL | PHY | BSc | EMP | MSc |  |
| N | 99 | 79 | 236 | 236 | 480 | 91 | 79 |  |
| Lower Whisker | 0 | 0 | 360 | 438 | 0 | 0 | 1036 |  |
| Upper Whisker | 7529 | 10878 | 10119 | 11184 | 10231 | 7693 | 12072 |  |
| P25 | 1813 | 2142 | 1964 | 2354 | 2177 | 1737 | 2262 |  |
| P50 | 2628 | 3573 | 3321 | 3798 | 3457 | 3006 | 4290 |  |
| P75 | 4100 | 6078 | 5226 | 5886 | 5399 | 4120 | 6186 |  |
|  |  |  |  |  |  |  |  |  |
| Sitting_minutes_per_day (Figure 3 in full text) | | |  | | |  |  |  |
|  | E_D | HEB | PFL | PHY | BSc | EMP | MSc |  |
| N | 110 | 100 | 282 | 269 | 567 | 101 | 93 |  |
| Lower Whisker | 120 | 90 | 0 | 30 | 0 | 120 | 120 |  |
| Upper Whisker | 960 | 900 | 900 | 750 | 900 | 960 | 540 |  |
| P25 | 420 | 300 | 300 | 300 | 300 | 360 | 240 |  |
| P50 | 480 | 420 | 405 | 360 | 420 | 540 | 300 |  |
| P75 | 668 | 540 | 540 | 480 | 540 | 675 | 360 |  |
|  |  |  |  |  |  |  |  |  |
| mMSD (Figure 4 in full text) | |  |  |  |  |  |  |  |
|  | E_D | HEB | PFL | PHY |  |  |  |  |
| N | 111 | 103 | 286 | 271 |  |  |  |  |
| Lower Whisker | 7 | 5 | 5 | 5 |  |  |  |  |
| Upper Whisker | 15 | 15 | 15 | 15 |  |  |  |  |
| P25 | 11 | 9 | 9 | 9 |  |  |  |  |
| P50 | 12 | 11 | 10 | 11 |  |  |  |  |
| P75 | 13 | 12 | 12 | 12 |  |  |  |  |
|  |  |  |  |  |  |  |  |  |
| Wake-Up Time (Figure 5 in full text) | | |  |  |  |  |  |  |
|  | BSc | EMP | MSc |  |  |  |  |  |
| N | 598 | 100 | 98 |  |  |  |  |  |
| Lower Whisker | 5.00 | 5.25 | 5.00 |  |  |  |  |  |
| Upper Whisker | 10.33 | 8.00 | 8.00 |  |  |  |  |  |
| P25 | 6.45 | 6.30 | 6.15 |  |  |  |  |  |
| P50 | 7.30 | 7.00 | 6.75 |  |  |  |  |  |
| P75 | 8.00 | 7.00 | 7.00 |  |  |  |  |  |
|  |  |  |  |  |  |  |  |  |

Multimedia Appendix 2: Calculated values for box and whisker plots of Figures 1 through 5 of the manuscript.


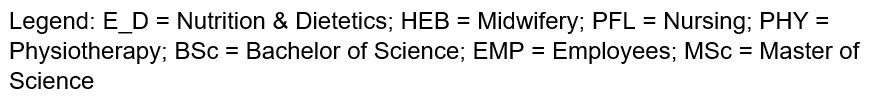

Supplement: Multimedia Appendix 2 [file publichealth_v7i4e26330_app2.docx]
